# A Rare Presentation of Acute Generalized Exanthematous Pustulosis With Refractory Shock

**DOI:** 10.7759/cureus.89386

**Published:** 2025-08-04

**Authors:** Maria C Perez-Mitchell, Mariela Ginés-Rosario, Ricardo Fernandez-Gonzalez, Jose J Gutierrez, Jenniffer Ramirez

**Affiliations:** 1 Pulmonary and Critical Care Medicine, San Juan City Hospital, San Juan, PRI; 2 Internal Medicine, San Juan City Hospital, San Juan, PRI; 3 Infectious Disease, San Juan City Hospital, San Juan, PRI

**Keywords:** acute generalized exanthematous pustulosis, circulatory shock, drug-induced adverse reaction, severe cutaneous adverse reaction, subcorneal pustule

## Abstract

Acute generalized exanthematous pustulosis (AGEP) is a severe cutaneous adverse reaction that presents with pustular lesions with underlying edematous and erythematous skin, accompanied by fever, leukocytosis, and neutrophilia. It is characterized by an abrupt onset, usually 24-48 hours after the inciting trigger. The incidence of AGEP is an uncommon skin reaction that is primarily seen in female patients. The clinical course is mostly limited to cutaneous findings, and only in rare cases, systemic involvement can occur. AGEP typically resolves within 1-2 weeks after discontinuation of the offending agent, and topical steroids are used for symptomatic relief. In more severe cases, systemic corticosteroids, vasopressors, or cyclosporine are useful, and vasopressors may be required in cases of circulatory shock. In this case, we present a middle-aged woman with fluid nonresponsive circulatory shock and acute kidney injury secondary to an uncommon presentation of AGEP, confirmed by histopathologic findings.

## Introduction

Acute generalized exanthematous pustulosis (AGEP) is a T-cell-mediated severe cutaneous adverse reaction that presents with sterile pustular lesions on an erythematous base and fever. It is attributed to drugs, such as antibiotics, in the majority of cases, and the condition can be self-limiting. AGEP is characterized by an abrupt onset, usually within 48 hours of ingesting the suspected agent [1]. Diagnosis can be made clinically with the support of histopathological findings. Typical histopathology features include the presence of intracorneal, subcorneal, or intraepidermal pustules and mixed perivascular and interstitial infiltrates [1]. Most cases resolved spontaneously after removal of the offending drug, but in severe cases, there can be mucous membrane involvement and systemic organ dysfunction [1]. AGEP has an estimated incidence of 3-5 cases per million per year, with systemic involvement seen in only 15-20% of patients, and while it often presents abruptly and extensively, it is generally self-limiting with a mortality of less than 5% [1]. 

## Case presentation

**Figure 1:**
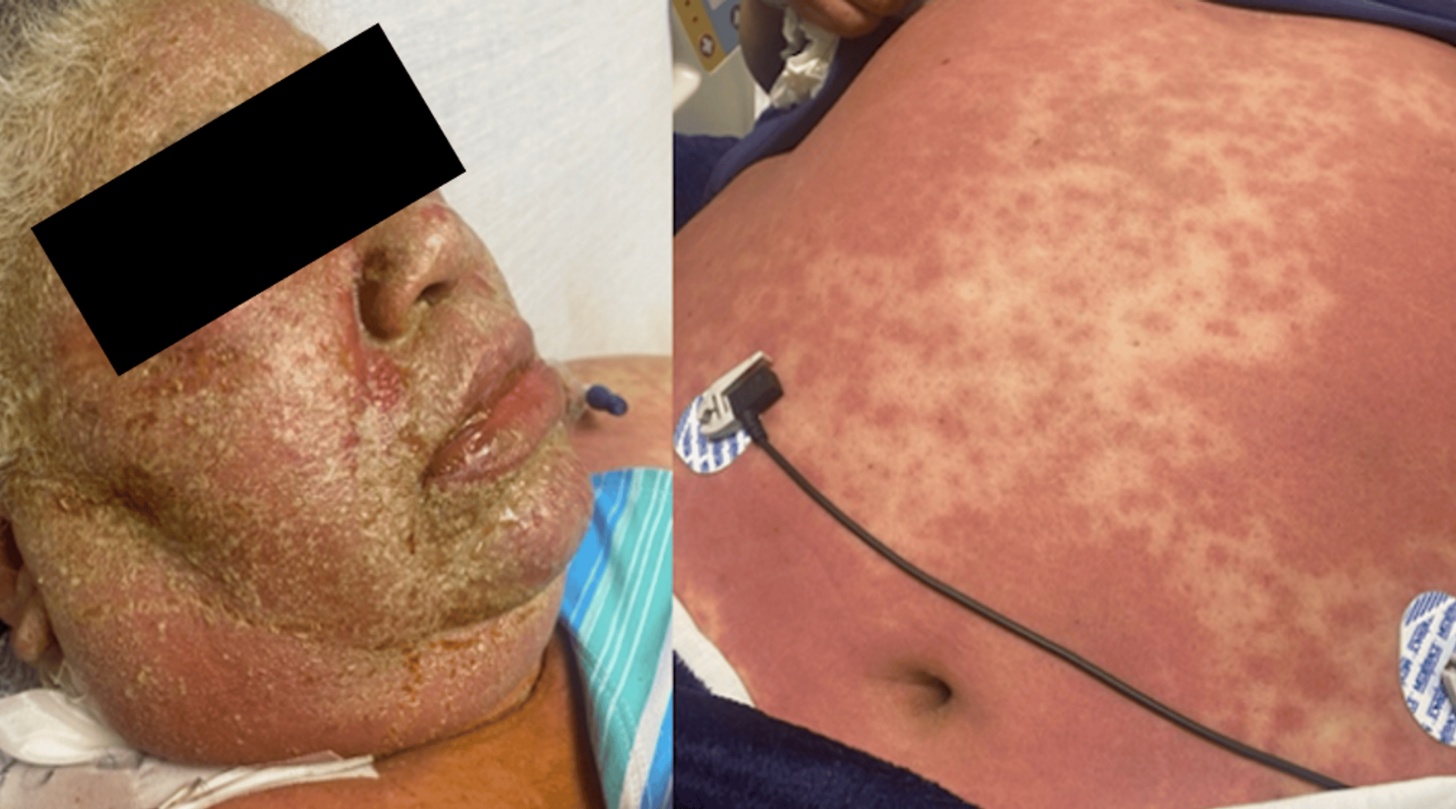
Acute medication-induced reaction presenting as pustules on an erythematous base in a cephalocaudal distribution.

**Figure 2:**
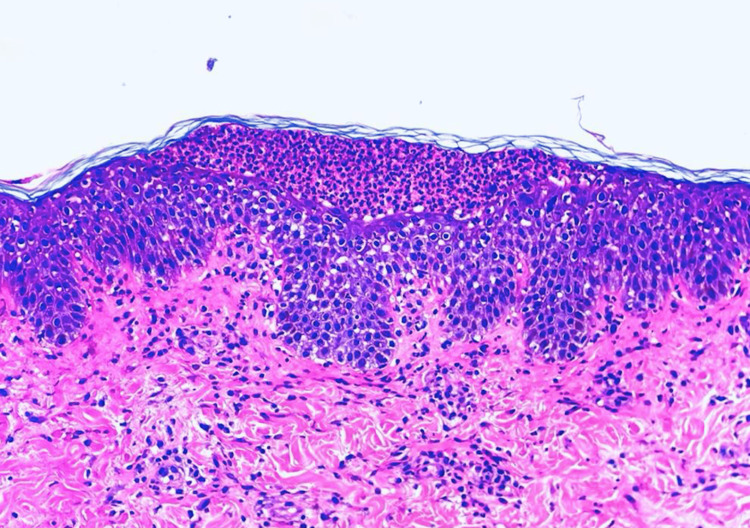
Histopathologic examination revealed a mixed-cell inflammatory infiltrate with eosinophils and subcorneal pustules.

A 60-year-old morbidly obese Hispanic woman with a past medical history of hypertension and chronic lymphedema presented to the emergency department complaining of a right lower extremity swelling with associated erythema of four weeks of evolution. The patient was diagnosed with sepsis secondary to non-purulent skin and soft tissue infection and cellulitis, which required hospital admission for intravenous antibiotics and intravenous hydration. The patient was admitted with intravenous clindamycin and ceftriaxone for 72 hours with no clinical improvement, which was then changed to ceftaroline for broad-spectrum coverage as a single agent. After 48 hours of treatment, the patient developed an acute rash described as sterile non-follicular pustular lesions on an erythematous base with multiple pustules over erythematous plaques that spread quickly from the face to the torso and upper extremities with sparing of the lower extremities, palms, and soles (Figure 1). It was accompanied by pruritus, facial edema, and a fever of 40 °C. No hemorrhagic crusting or mucous membrane involvement was present. Nikolsky’s sign was negative. Oral and genital mucosa were carefully examined and found to be uninvolved. Hemodynamics was stable at that moment. She required only a nasal cannula 2 L for mild hypoxemia. Naranjo's adverse drug reaction probability scale was 6, which means a high probability of the reaction after the antibiotic use. The cutaneous reaction was managed as an anaphylactic shock with rash with intramuscular epinephrine every five minutes and adjunctive treatment with antihistamines and intravenous corticosteroids. After two hours of observation, the patient became hypotensive and unresponsive to intravenous hydration and medical management. Airway and breathing were not compromised, but circulatory support was required. Refractory shock was managed as an anaphylactic reaction with intravenous epinephrine to maintain mean arterial pressure and perfusion state. Management with intravenous epinephrine, corticosteroids, and antihistamines continued in the ICU, with adequate response, confirming anaphylaxis as the cause of shock. As part of the workup, multiple blood cultures turned out negative, and laboratories were remarkable for an acute kidney injury, leukocytosis with neutrophilia, and elevated C-reactive protein levels, which favors an acute event (Table 1). In comparison to typical AGEP presentations, which often feature leukocytosis and modest inflammatory marker elevation, our patient exhibited marked CRP elevation and acute kidney injury, findings that are less commonly reported and emphasize the atypical severity of this case. Dermatology was consulted for skin biopsy due to suspected AGEP. The other principal noninfectious differential diagnoses included drug rash with eosinophilia and systemic symptoms, toxic epidermal necrosis, Stevens-Johnson syndrome, and pustular psoriasis. While waiting for the results of the histopathology, diagnosis of AGEP was made based on clinical features and onset of events after 24-48 hours of ceftalorine administration. It was confirmed with histopathology, which showed mixed-cell inflammatory and perivascular infiltrates with eosinophils and the presence of subcorneal pustules, which is AGEP’s hallmark (Figure 2). Further stratification was made based on elevated CRP levels and systemic involvement, which are an uncommon presentation in AGEP. Due to systemic involvement and circulatory shock, the patient received intravenous epinephrine for three days until hemodynamics stabilized. AGEP was treated with high-potency topical steroids with clinical improvement. Her rash began to clear with desquamation, and she was discharged home stable. Stratification was made with laboratories, which in our case presented with revealed leukocytosis with predominance of neutrophils, acute kidney injury and elevated CRP levels, which are uncopresentationsation in AGEP. Acute kidney injury and hypotension resolved after two days of intravenous hydration, intravenous epinephrine, and topical corticosteroids. Upon follow-up, resolution is marked by desquamation.

**Table 1:** Laboratory parameters for AGEP. AGEP: Acute generalized exanthematous pustulosis

Laboratory Tests	Result	Reference Range
White blood cell count (x10^3/uL)	17.4	(4.8 - 10.8)
Neutrophil count (%)	84.1	(42.2 - 75.2)
Creatinine (mg/dL)	1.7	0.60 -1.10
Blood urea nitrogen (mg/dL)	31	8.0 - 21.0

## Discussion

AGEP is a rare drug-induced reaction that must be suspected with an abrupt onset of pustular cutaneous lesions and associated fever [2]. The typical clinical picture and course of AGEP are confirmed with histopathological findings [3]. The most frequent culprits include antibiotics (most common) such as beta-lactams, aminopenicillins (e.g., ampicillin, amoxicillin), and cephalosporins (e.g., ceftriaxone, cefazolin), among other drugs. Characteristically, patients with AGEP develop these cutaneous features with no systemic involvement, but in 15% of this population, acute kidney injury, liver failure, or hemodynamic instability can occur [3,4]. In the case of multiple organ dysfunction, circulatory or respiratory compromise, a patient with AGEP should be treated under intensive care. The resolution of the rash occurs spontaneously within 1-2 weeks after discontinuation of the offending agent and can be treated with topical steroids [4,5]. The mortality is 5% if diagnosed promptly [1] and no systemic involvement is present. AGEP recurs with the reintroduction of the causative drug [4,5]. The culprit agent can be confirmed with a positive skin patch test.

## Conclusions

AGEP is a severe pustular skin manifestation that can be caused by medications. AGEP must be diagnosed early to avoid severe and life-threatening complications. Adverse cutaneous drug reactions are widely acknowledged as significant global health issues, placing a substantial burden on healthcare systems. Physicians should be aware of specific red flags to rapidly identify these cutaneous drug eruptions to initiate appropriate treatment. This case highlights that AGEP remains an underreported condition, underscoring the need for heightened clinical awareness, timely recognition, and thorough documentation to improve diagnosis and management.
